# Expert elicitation database capturing diversity and cultural drivers of food choice and nutritional implications in eastern India

**DOI:** 10.1016/j.dib.2020.106330

**Published:** 2020-09-23

**Authors:** Marie Claire Custodio, Jhoanne Ynion, Rosa Paula Cuevas, Arindam Samaddar, Anindita Ray (Chakravarti), Suva Kanta Mohanty, Matty Demont

**Affiliations:** a International Rice Research Institute, Los Baños, Laguna, Philippines; b Department of Food & Nutrition, Maharani Kasiswari College, University of Calcutta, India; c Kalinga Institute of Industrial Technology, KIIT School of Management, Bhubaneswar, India; d Institute of Rural Management Anand (IRMA), Anand, Gujrat, India

**Keywords:** Gastronomic systems research, Expert elicitation, Food choice, Nutrition, Eastern India

## Abstract

Two expert elicitation workshops were conducted in 2017 to capture the diversity and cultural drivers of food choice of low- and middle- income households in the states of West Bengal and Odisha in eastern India. Experts representing the fields of nutrition, home science, food technology, and food service industry were invited to participate. Following the “gastronomic systems research” framework, the food experts determined the eating occasions, dishes and ingredients that would culturally define the target population in their respective states. To zoom in further on the nutritional implications, one of the two states was selected for further in-depth study by expanding the list of dishes and conducting nutritional analysis. The approach is elaborated in the article “Capturing diversity and cultural drivers of food choice in eastern India” [1]. The workshop generated two databases: (i) “List of dishes and ingredients from expert elicitation workshop” and (ii) “Database of eastern Indian dishes”. The former was used to differentiate the eating occasions based on dishes, the proportion of dishes based on dish classification, and the dietary diversity score of each occasion. The dietary diversity score was then used to analyze the nutritional composition of dishes in terms of three macro nutrients such as protein, carbohydrates and fat in each eating occasion. The databases provide a useful baseline for nutritionists, policymakers, and food system actors to design nutrition intervention strategies for the purpose of developing planetary health diets in eastern India.

## Specifications Table

**Table:** 

Subject	Social Sciences (General)
Specific subject area	Expert elicitation used to capture the diversity and cultural drivers of food choice and nutritional implications [2,3]
Type of data	Table
How data were acquired	The data were acquired through expert elicitation workshops in the states of Odisha and West Bengal in eastern India, following the Gastronomic Systems Research (GSR) framework [2,3]. In each expert elicitation workshop, the food experts were tasked with specific exercises to identify eating occasions, common dishes for each occasion, and ingredients that would be typically consumed by the target population following a standard structure of individual, intra-team, and inter-team elicitation methods. To estimate the nutritional composition, the dishes that made the final list were standardized and prepared in a food laboratory. Each dish was then classified into a food group according to its most prominent ingredient: starches, vegetables, non-vegetable (i.e., meat, fish, egg), pulses, fruits, and dairy.
Data format	Raw Analyzed
Parameters for data collection	Experts from the fields of study on nutrition, home science, food technology, and food service industry were invited to the workshops. They were identified mainly based on overall knowledge about the food consumption and culture of the target population in the state where they practice their professions.
Description of data collection	Social scientists from the International Rice Research Institute facilitated the expert elicitation workshops, following the GSR framework [2] and guided by a protocol [3]. The basic approach of the process was to facilitate system-level thinking, active engagement in individual- and group-level discussions, encourage out-of-the box thinking, and consensus building. West Bengal was then selected for further in-depth study and the expert from University of Calcutta refined the list of dishes captured by the experts in the Kolkata workshop and conducted the nutritional analysis.
Data source location	Country: India City/Town/Region: Kolkata, West Bengal and Bhubaneswar, Odisha
Data accessibility	In a public repository:Repository name: Harvard Dataverse List of dishes and ingredients from expert elicitation workshop [4]: • Data identification number: https://doi.org/10.7910/DVN/CRQFBO • Direct URL to data: https://dataverse.harvard.edu/dataset.xhtml?persistentId=doi:10.7910/DVN/CRQFBO Database of eastern Indian dishes [5]: • Data identification number: https://doi.org/10.7910/DVN/SRM5GA • Direct URL to data: https://dataverse.harvard.edu/dataset.xhtml?persistentId=doi:10.7910/DVN/SRM5GA
Related research article	Samaddar, A., Cuevas, R. P., Custodio, M. C., Ynion, J., Ray (Chakravarti), A., Mohanty, S. K. and Demont, M. Capturing diversity and cultural drivers of food choice in eastern India. *International Journal of Gastronomy and Food Science*, 22 (2020) 100249. DOI: 10.1016/j.ijgfs.2020.100249

## Value of the Data

• The success of encouraging a shift towards “planetary health diets”, diets that are low in animal-sourced food and are mainly plant-based [6], depends on consumers’ culture, socio-cultural context, and the food environment. Expert elicitation is a useful first step to capture diversity and cultural drivers of food choice of a target population and to identify nutrition interventions towards healthier diets.
• The Gastronomic Systems Research (GSR) framework conceptualizes food choice as a hierarchical system of eating occasions that are defined by context, culture and the food environment. Eating occasions command a set of dishes, which in their turn command certain ingredients and ingredient pairings with specific cooking and sensory quality attributes and nutritional content. The GSR framework helps gain a better understanding of who, where, when, what, and why people eat. It also unveils possible entry points for nutrition interventions that can assist nutritionists, policy makers and food system actors in designing nutrition intervention strategies [1], [2], [3].
• The databases generated in the expert elicitation workshops can be used to design targeted questionnaires for consumer surveys in a second stage and aid the design of behavioral experiments and interactive food choice tablet applications as a third stage to elicit consumer behavior [7].
• The “list of dishes and ingredients” database provides indications of preferences of food habits across urban and rural contexts. The dish classifications based on ingredients provide indications of dietary diversity per occasion. This information provides a first glimpse of nutrition diversity in the different occasions, which may be used to design nutrition interventions that are based on eating occasions.
• The “database of eastern Indian dishes” captures information on the macronutrient content of West Bengal dishes. This provides insights on nutritional implications of these food choices for low- and middle-income households.

## 1 Data Description

The first database comprises the list of dishes and ingredients from the expert elicitation workshops in both states in eastern India [4]. The workshop in Bhubaneswar, Odisha was held on 18 July 2017 and the workshop in Kolkata, West Bengal was held on 21 July 2017. In each state, the food experts (i.e., nutritionists, food scientists, home scientists, restaurant holders, and chef) identified a maximum of 20 typically consumed dishes per occasion—breakfast, morning snacks, lunch, afternoon snacks, dinner, and special occasions. These dishes reflect the diversity and cultural drivers of food choice of the target population—households in the low- and middle-income groups in urban and rural West Bengal and Odisha [1]. A dish is any food prepared in a specific way composed of component ingredients. A dish may contain a single component (e.g., rice) or multiple components. Desserts were considered as dishes. Beverages were not included in this study.

After the workshops in Bhubaneswar, Odisha and Kolkata, West Bengal, the latter was then selected for further in-depth study and one of the experts from Kolkata refined the list of dishes captured in the workshop and conducted the nutritional analysis with the output captured in the second database [5]. The second database consists of the more refined list of dishes in West Bengal with ingredients and nutritional content (i.e., total energy, carbohydrates, protein, and fat). These dishes are typically consumed by households which belong to urban and rural low- and middle-income groups in West Bengal. The second database provides a first glimpse of the nutrition diversity of the different occasions [1].

## 2 Experimental Design, Materials and Methods

### 2.1 Expert elicitation

We conducted two expert elicitation workshops in eastern India. We invited experts from four different professional backgrounds: nutrition, home science, food technology, and foodservice industry (i.e., chefs and restaurateurs). These experts were selected based on their educational attainment, subject knowledge (e.g., regional specificity of food preparation techniques, consumer preferences), prior work experience in food research, willingness to collaborate, and overall knowledge about the food consumption and culture of the target population in the state where they practice their professions. Chefs and restaurateurs were identified who catered mainly to the target population (i.e., low- and middle-income households) in the states in which they operate. They were selected based on their interest, availability, length of experience, and knowledge of the food culture in the state where their restaurants were located. They were compensated for their transport and participation.

A total of 17 experts accepted the written invitations and gave their consent for participating in the workshop. Nine of the participants were nutritionists and home scientists, four were food technologists, three were restaurant owners, and one was a chef. The majority of the nutritionists, home scientists and food technologists have PhD degrees in their respective fields of specialization. The nutritionists were working in various work environments, for example one of them was a university professor, another was a dietician working in a hospital, and another was a school teacher. The home scientists who participated in the workshops worked in a University, Krishi Vigyan Kendra (KVK), and an NGO with wide experience working with rural and urban populations. The food technologists who participated in the workshops were working in universities and in the private sector as freelancers. The chef and restaurant owners were well exposed to West Bengal and Odisha's food culture, cooking methods, ingredients used, consumer preferences, and demand for specific food choices across urban and rural populations. The restaurant owners operate several store branches in both states.

In each expert elicitation workshop, experts were first requested to define the target population—low- and middle-income households in the state where they practice their professions—to improve the context-specificity of food choices. After being presented with secondary income data [8], the experts were requested to reach consensus on a cut-off point for the lower and upper limits of the income range of the targeted population irrespective of its urbanity. The experts were then introduced to the GSR framework and specific examples were provided to ensure full understanding of the context-specificity of its hierarchical structure [2]. Participants were tasked with specific exercises to identify eating occasions, common dishes for each occasion, and ingredients that would be typically consumed by the target population throughout the year following a standard structure of individual, intra-team, and inter-team elicitation methods [1]. During the first exercise, the experts identified the eating occasions of the target population. In the next stage, the experts were separated into two teams (consisting of representatives from each discipline) and the occasions were divided equally among the teams. The participants, individually and as a group, identified the most common dishes (N = 20) consumed during each assigned occasion by the target population in their state. The list of dishes was reviewed and refined during the inter-group consultations. Afterwards, each team finalized their lists and presented the final output for the next exercise: identification of the ingredients in each dish.

The dishes identified by the experts were classified into six food groups based on central or dish-defining ingredients: (i) starch (includes grains, roots and tubers, and plantains); (ii) pulses, nuts, and seeds (including beans, peas, lentils, peanuts, and soybeans); (iii) vegetarian (includes dark green leafy vegetables, and all other vegetables); (iv) non-vegetarian (includes meat, poultry, eggs, and fish); (v) fruit (including those rich in Vitamin A); and (vi) dairy. Such classification is a simplification of the ten food groups used to determine the dietary diversity scores for women [9]. The entire gastronomic system for each state was captured in a database which provides a baseline of current diversity and cultural drivers of food choice in eastern India.

### 2.2 Nutritional analysis

The list of dishes was reviewed to ensure that it represents dishes consumed by the target population and to assess the extent to which the dishes could be interchanged according to the occasion. Each dish could have regional recipe variants; these variants were obtained through an informal structured survey within Kolkata. The city was divided into four zones (north, east, south, and west) based on the Kolkata Municipal Corporation (KMC) map. Thirty households from each zone were randomly selected. During the survey, the households detailed their food habits and recipes of dishes that they had mentioned.

To estimate the nutritional composition, the dishes that made the final list were standardized and prepared in the laboratory of the Department of Food & Nutrition. The most common recipe was considered in creating the benchmark version of the dish. Each dish was then classified into a food group according to its most prominent ingredient: starches, vegetables, non-vegetable (i.e., meat, fish), pulses, fruits, and dairy. The adult portion size of each dish was calculated by dividing the amount (by weight) of food prepared for a specific occasion by a household in the target population composed of two adults and two children by the household size (standardized household size = 4). Based on the recipe previously benchmarked, the typical amount of ingredients used by the target population could be determined. Such an approach considered that portion sizes could vary by occasion. Then, the dishes that were typically prepared at home were cooked as per the benchmarked recipe, while dishes that were typically not prepared at home were purchased ready-made in the market, such as retail stores and food stalls. Finally, the macronutrient composition (i.e., carbohydrates, protein, and fat) and the total calories of each dish were estimated using the Indian Food Composition Table [10]. All data were stored in a database [5].

### 2.3 Data curation

To reduce sparsity, name variants of dishes identified by the experts during the elicitation workshops were classified into one group (e.g., “Ghuguri”, “Ghoogni”, and “Ghugni” were all grouped as “Ghugni”). The text data was then stored in a relational database created in MySQL Workbench (Version 8.0.13). Likewise, the data obtained from the nutritional analyses of benchmark dishes in Kolkata were stored in a MySQL database.

## Ethics Statement

The expert elicitation workshops were organized under the “Behavioral drivers of food choice in eastern India” project. The research protocol of this project was reviewed and approved by the Drivers of Food Choice (DFC) Competitive Grants Program, which is managed by the University of South Carolina, Arnold School of Public Health, USA. The behavioral experiments that were guided by the results of the expert elicitation workshops obtained ethics approval from IRRI's Institutional Research Ethics Committee (IREC 18-001). However, at the time the workshops were conducted, there were no clear guidelines on the need for ethics approval by a local research ethics committee for this type of expert elicitation following: (i) the 2007 Indian Council of Medical Research National Ethical Guidelines for Biomedical and Health Research Involving Human Participants; and (ii) the 2000 National Committee for Ethics in Social Science Research in Health (NCESSRH) Ethical Guidelines for Social Science Research in Health.

We believe that this workshop was conducted in alignment with the key principles and ethical standards laid down in the 1964 Declaration of Helsinki and that it addressed an issue of national importance for India. The experts who participated in the workshop are all educated and technical experts in the field of nutrition research, food technology and food service industry. The collective discussions around food habits, diets and nutrition interventions were not perceived to be sensitive and were considered to be part of their daily routine work. Before the start of the workshop, the facilitators presented the objective and aims of the workshop and how the collective information provided by the experts would be used to inform the present study on food choice. The experts accepted the written invitations and gave their consent for participating in the workshop. They were compensated for their transport and participation. One of the experts proposed to conduct a further in-depth nutritional analysis and was invited to become a co-author of this study. All experts were mentioned by name and affiliation and acknowledged in the manuscript. The databases generated from the expert elicitation workshops are publicly available at Harvard Dataverse [4].

## CRediT authorship contribution statement

**Marie Claire Custodio:** Writing - original draft, Writing - review & editing, Project administration. **Jhoanne Ynion:** Investigation, Data curation, Visualization, Project administration. **Rosa Paula Cuevas:** Conceptualization, Methodology, Investigation, Data curation, Visualization. **Arindam Samaddar:** Investigation, Validation, Writing - review & editing, Project administration. **Anindita Ray (Chakravarti):** Investigation, Data curation, Writing - review & editing, Validation, Resources. **Suva Kanta Mohanty:** Investigation, Writing - review & editing, Validation, Resources, Project administration. **Matty Demont:** Conceptualization, Methodology, Investigation, Writing - review & editing, Supervision, Funding acquisition.

## Declaration of Competing Interest

The authors declare that they have no known competing financial interests or personal relationships which have, or could be perceived to have, influenced the work reported in this article.

## Appendix Supplementary materials

Image, application 1
